# Surgical interventions for empty nose syndrome: A meta-analysis of meta-analyses

**DOI:** 10.1007/s00405-025-09501-x

**Published:** 2025-06-03

**Authors:** Leire Aguirre-Peña, Octavio Garaycochea, Isam Alobid, Holger Sudhoff, Manuel Bernal-Sprekelsen

**Affiliations:** 1 University of Barcelona Medical School, Villaroel 170, Esc,.8, 2 A, 08036 Barcelona, Spain; 2 ENT-Dept, Clinica Univ.de Navarra, Pamplona, Navarra Spain; 3 Instituto de Investigaciones Biomédicas August Pi I Sunyer (IDIBAPS), Barcelona, Spain; 4 ENT-Dept. Hospital Clinic, Barcelona, Spain; 5 Kopfzentrum Bielefeld, Bielefeld, Germany

**Keywords:** Empty nose syndrome, Turbinate surgery, Quality of life, Patient-reported outcome measures, Sino-Nasal Outcome Test (SNOT)

## Abstract

**Introduction:**

Empty nose syndrome (ENS) is a complex iatrogenic condition resulting from excessive inferior turbinate resection, leading to paradoxical nasal obstruction despite an objectively patent airway.

**Methods:**

This system review and meta-analysis of meta-analyses, performed with the AMSTAR2 tool, evaluates surgical treatment outcomes for ENS, focusing on validated patient-reported measures such as the ENS6Q and SNOT questionnaires.

**Results:**

Data from three meta-analysis, including over 1500 cases, highlights the long-term benefits and limitations of various surgical approaches.

**Discussion:**

While surgical interventions demonstrate sustained improvements in patient quality of life, substantial heterogeneity across meta-analyses and overlapping primary data highlight the need for standardized protocols, harmonized outcome measures, and meta-evaluation frameworks in future ENS research.

## Introduction

Empty nose syndrome (ENS) constitutes a debilitating condition characterized by paradoxical nasal obstruction, dryness, and impaired nasal function following turbinate resection [1,2]. Its pathophysiology remains controversial, involving anatomical, physiological, and neurological factors [3]. ENS patients frequently experience severe discomfort, including nasal crusting, a suffocating sensation, and hypersensitivity to airflow, significantly impairing their quality of life [4]. ENS *is rare, with an estimated prevalence of 0.05–0.1% among post-turbinate resection patients, but likely underreported due to misdiagnosis and lack of awareness* [5].

ENS is associated with elevated rates of anxiety, depression, and suicidal ideation, with psychological symptoms often outlasting physical recovery. Studies report up to 70% of ENS patients experience clinically significant distress [6–8]. Therefore, comprehensive assessment must consider both physiological and psychological domains.

### Diagnosing

ENS remains challenging due to the absence of universally accepted objective criteria. The ENS6Q, a validated 6-item scale with a diagnostic threshold of ≥11, quantifies symptom severity from 0 (none) to 5 (severe) for each item [9]. Additional assessments include the"Cotton Test,"which evaluates symptom improvement following temporary nasal augmentation, and the Sino-Nasal Outcome Test (SNOT), which measures the broader impact of sinonasal conditions on quality of life[9,10].

While conservative treatments such as nasal hydration and humidification offer only temporary relief, surgical intervention remains the primary approach for managing refractory ENS[11]. Inferior meatus augmentation procedures (IMAP) using autologous or synthetic grafts are commonly performed, with emerging techniques such as stem cell-enriched fat grafts showing promise for tissue regeneration[12,13]. However, further investigation is needed to establish the long-term efficacy and safety of these procedures.

Objectives: This meta-analysis of meta-analyses aims to:

1. Assess the effectiveness of surgical interventions for ENS based on validated outcome measures (ENS6Q and SNOT).
2. Evaluate heterogeneity in the existing literature and identify research gaps.
3. Provide recommendations for optimizing ENS treatment strategies.

### Materials and methods: Meta-analysis of meta-analyses

A systematic literature review was conducted following PRISMA guidelines. Studies published between January 1, 2013, and September 1, 2024, *in PubMed, Embase, and Cochrane CENTRAL* were identified using the following search terms: ("Empty Nose Syndrome [Title/Abstract]"OR"ENS [Title/Abstract]") AND ("Treatment [Title/Abstract]"OR"Management [Title/Abstract]"OR"Surgery [Title/Abstract]"OR"Surgical [Title/Abstract]").

### Inclusion and exclusion criteria

Inclusion**:** Studies on surgical ENS treatment using ENS6Q or SNOT as outcome measures, including systematic reviews, meta-analyses, and prospective studies.

Exclusion: Studies lacking validated outcome measurements, case reports, and non-English publications.

To prevent duplication bias, only meta-analyses were included in final synthesis. Eight eligible primary studies were excluded from quantitative synthesis and qualitatively discussed.

### Selection process

A total of 497 studies were screened, with 11 meeting the inclusion criteria. These included 8 primary studies and 3 meta-analyses[14–24]. To prevent data duplication, only meta-analyses were included in the final synthesis. The quality of included meta-analyses was evaluated using AMSTAR 2 by two independent reviewers. Discrepancies were resolved by consensus [25].

## The study selection process is summarized in Figure 1 (PRISMA Flow Diagram).

**Fig. 1:**
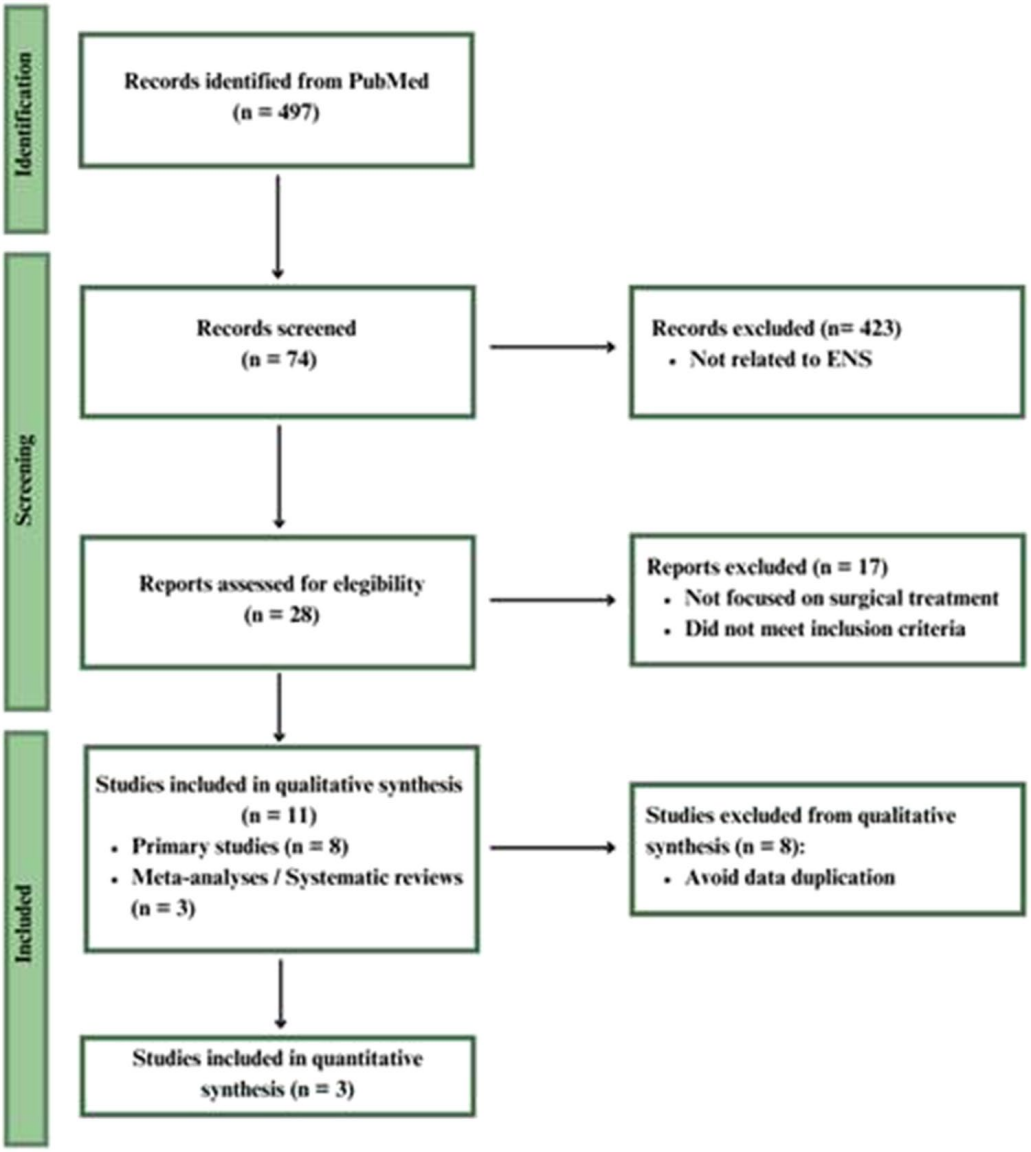
PRISMA Flow diagram for study selection

### Statistical analysis

A meta-analysis was performed, integrating aggregated data while adjusting for duplicated studies. Mean differences (MD) and standardized mean differences (SMD) were calculated for ENS6Q and SNOT scores at 1, 3, 6, and 12 months post-surgery. Heterogeneity was assessed using the I [2] statistic. A random-effects model was applied to account for between-study heterogeneity. Heterogeneity was assessed using I^2^ with conventional interpretation: low (<25%), moderate (25–50%), and high (>75%).

This review was not registered in PROSPERO but adhered to PRISMA guidelines. This study did not require IRB approval as it utilized only previously published data.

Publication bias was not formally assessed due to the small number of meta-analyses, precluding funnel plot analysis or Egger’s test

Based on prior studies, a change >8 points in ENS6Q is considered clinically meaningful (Velasquez et al., 2017). Similarly, a standardized mean difference (SMD) >1.0 in SNOT is regarded as a substantial improvement in sinonasal-related quality of life.”

## Results

Three meta-analyses were included after screening 497 studies (Table 1). Eight primary studies were excluded from quantitative synthesis to avoid duplication.

**Table 1:** Summary of Included Meta-Analyses on ENS Surgical Outcomes

Author	Year	No. of Patients	Type of Graft	Follow-Up Duration	Outcomes Measured	AMSTAR 2 Rating
Hussain S. et al. [22]	2024	733	Autologous (Fat, Dermis, Fascia)	1–12 months	ENS6Q, SNOT	Moderate
Kim DH et al. [13]	2024	512	Mixed (Fat, Medpor)	1–12 months	ENS6Q, SNOT	Low
Ma ZX et al. [24]	2017	283	Synthetic (Medpor, Silastic)	3–12 months	SNOT	Critically Low

A total of 1,528 patients undergoing surgical intervention for ENS were included. Different augmentation materials, including autologous and synthetic grafts, were used.

Baseline age ranged from 26–56 years; approximately 60% were male. Graft types included fat, fascia, dermal matrix, and silastic.

The AMSTAR 2 appraisal revealed variable quality across included meta-analyses, with two rated as ‘moderate’ and one as ‘low’ confidence in findings.”

Overlapping studies across MAs were identified by author/year cross-checking; duplicate data were removed from pooled calculations where applicable.

Subgroup analysis by graft type and follow-up duration was not possible due to inconsistent stratification across studies.

### ENS6Q outcomes

**Fig. 2:**
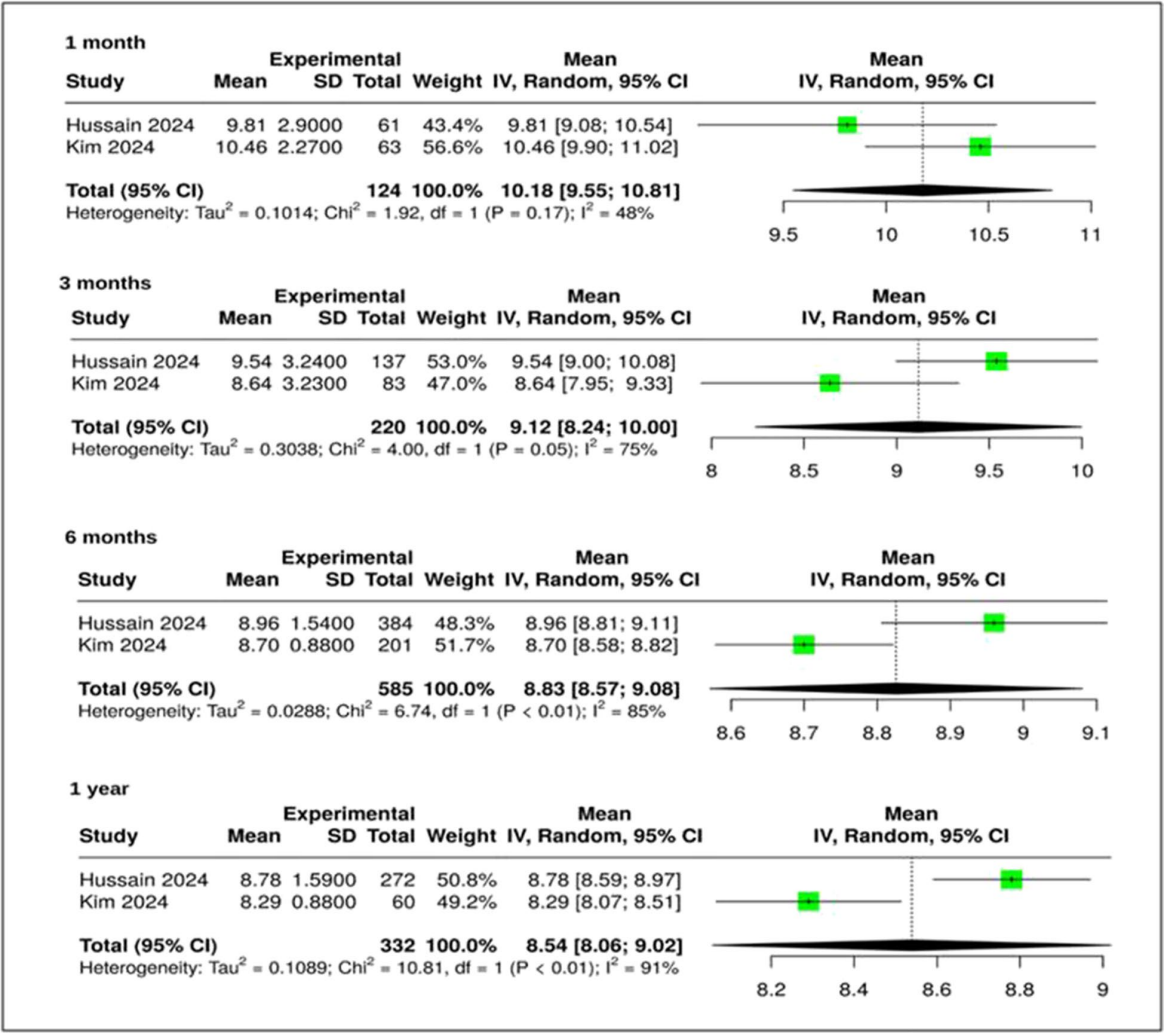
Forest plot of ENS6Q displaying pooled mean differences (MD) outcomes at 1, 3, 6, and 12 months post-surgery (MD = mean differences, 95% CI, and I^2^ values)

Two meta-analyses reported significant reductions in ENS6Q symptom severity (Figure 2**)**

$$\begin{array}{c}1\text{ month}:\text{ mean differences }(\text{MD}) = 10.18 (95\text{\% confidence interval }(\text{CI}): 9.55, 10.81); {\text{I}}^{2} = 48\text{\%}\\ 3\text{ months}:\text{ MD }= 9.12 (95\text{\% CI}: 8.24, 10.00); {\text{I}}^{2} = 75\text{\%}\\ \begin{array}{c}6\text{ months}:\text{ MD }= 8.83 (95\text{\% CI}: 8.57, 9.08); {\text{I}}^{2} = 85\text{\%}\\ 12\text{ months}:\text{ MD }= 8.54 (95\text{\% CI}: 8.06, 9.02); {\text{I}}^{2} = 91\text{\%}\end{array}\end{array}$$

At 1, 3, 6, and 12 months postoperatively, pooled mean differences (MD) in ENS6Q scores across meta-analyses showed statistically significant and clinically meaningful improvements, with all estimates exceeding the 8-point threshold typically associated with symptomatic relief. At 12 months, the pooled MD was 8.54 (95% CI: 8.06–9.02), as shown in Figure 2. These results reflect consistent symptom improvement, although heterogeneity ranged from moderate to high (I^2^ = 48–91%).

### SNOT outcomes

**Fig. 3:**
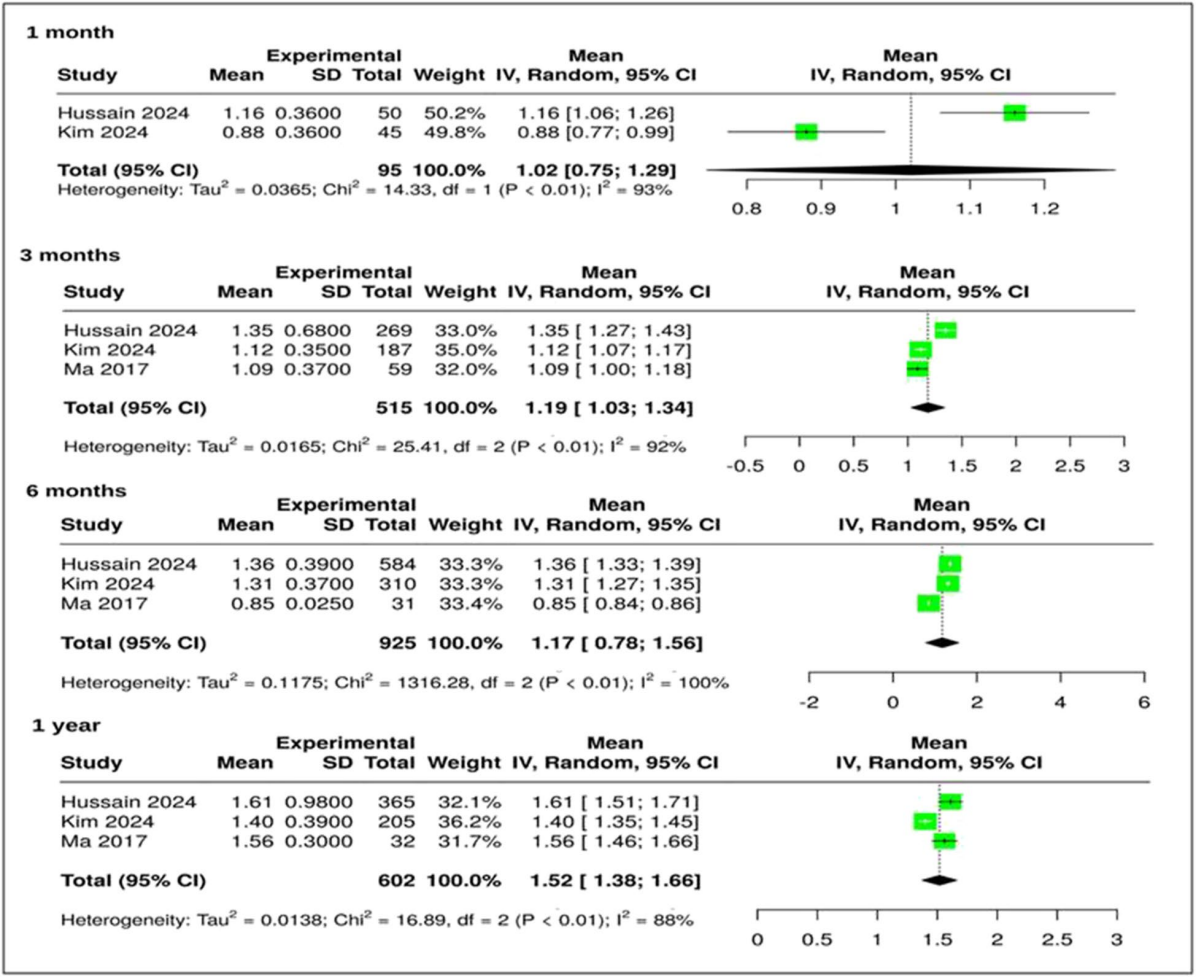
Forest plot of SNOT outcomes showing pooled standardized mean differences (SMD) and 95% confidence intervals (CI) at multiple timepoints and I^2^ values indicating heterogeneity

All three meta-analyses reported significant improvements in SNOT scores **(**Fig. 3)

$$\begin{array}{c}1\text{ month}:\text{ standardized mean differences }(\text{SMD}) = 1.02 (95\text{\% CI}: 0.75, 1.29); {\text{I}}^{2} = 93\text{\%}\\ 3\text{ months}:\text{ SMD }= 1.19 (95\text{\% CI}: 1.03, 1.34); {\text{I}}^{2} = 92\text{\%}\\ \begin{array}{c}6\text{ months}:\text{ SMD }= 1.17 (95\text{\% CI}: 0.78, 1.56); {\text{I}}^{2} = 100\text{\%}\\ 12\text{ months}:\text{ SMD }= 1.52 (95\text{\% CI}: 1.38, 1.66); {\text{I}}^{2} = 88\text{\%}\end{array}\end{array}$$

Improvements in SNOT scores were also evident at all follow-up intervals. Standardized mean differences (SMD) exceeded the accepted threshold of ≥1.0 for clinically significant improvement in sinonasal quality of life. At 12 months, the pooled SMD was 1.52 (95% CI: 1.38–1.66), as shown in Figure 3. These findings indicate large effect sizes and sustained benefits, despite high heterogeneity (I^2^ = 88–100%).

## Discussion

### Surgical outcomes and interpretation

This meta-analysis of meta-analyses confirms consistent postoperative improvements in ENS6Q and SNOT scores over 12 months, reflecting both symptom relief and psychosocial benefit.

ENS6Q scores show a gradual decline in symptom severity over time, whereas SNOT scores indicate progressive improvement. This reflects the difference in focus between ENS6Q (ENS-specific symptoms) and SNOT (broader sinonasal health and psychosocial well-being)[10,11]. Long-term quality-of-life improvements may be influenced by psychological adaptation and behavioral adjustments rather than structural restoration alone.

ENS6Q improvements >8 points and SNOT SMD >1.0 are considered clinically meaningful, based on previous literature [26].

### Challenges and limitations

Despite promising results, significant heterogeneity (I^2^ = 48–100%) across studies for ENS6Q Outcomes underscores the need for methodological consistency in ENS research. This heterogeneity is likely due to differences in surgical technique, graft type, outcome measures, and patient selection.

One major challenge is that ENS6Q and SNOT are subjective measures that lack objective physiological assessments, such as airflow dynamics and imaging. Notably, while SNOT-22 has not been specifically validated for ENS, it is commonly used to assess symptoms related to the nose and paranasal sinuses. Additionally, variability in surgical techniques, patient selection, and follow-up durations limits the generalizability of findings. Furthermore, the scarcity of psychosocial data highlights the need for further research into mental health outcomes following surgery.

Added limitations are the subjective outcome reliance, the lack of objective measures (e.g., rhinomanometry), short follow-up duration in some included studies and no publication bias or sensitivity analysis.

Funnel plots/Egger’s tests were not performed due to study number constraints.

Subgroup analyses (e.g., graft type, follow-up) were not feasible due to data stratification.

### Psychological outcomes in ENS surgical studies: Narrative summary

Several of the studies referenced in the included meta-analyses report significant psychological burden among ENS patients, both pre- and post-surgery:

Kim et al.[7] found that ENS patients experienced significantly higher stress levels compared to patients with chronic rhinosinusitis or allergic rhinitis. Psychological distress scores were significantly elevated on validated stress scales, with >60% meeting clinical thresholds for moderate to severe stress**.** Huang et al.[8] **r**eported that **up** to 28% of ENS patients had suicidal ideation**,** and nearly 70% exhibited signs of clinical depression or anxiety**,** emphasizing the psychiatric risk profile in this population**.**

Huang et al., 2023 [5] highlighted sleep impairment as a frequent co-morbidity, often associated with psychological distress and reduced quality of life, and found persistent improvement post-surgery in both nasal symptoms and sleep quality.

While primarily focused on surgical outcomes via ENS6Q and SNOT, Kim et al., 2024 [13] the authors reported improvements in general well-being and emotional domains post-intervention, although no formal psychiatric scales were used.

### Future directions

Standardizing ENS surgical interventions requires unified protocols and objective evaluation metrics to reduce heterogeneity and improve clinical reliability. Longitudinal studies with extended follow-ups are essential to assess the long-term effectiveness of different procedures, as symptom improvement may fluctuate over time.

Integrating psychosocial support into postoperative care is equally important, as many ENS patients experience anxiety and depression, affecting their overall well-being. Future research should combine psychological and physiological assessments to refine surgical techniques and enhance long-term patient outcomes.

Future studies should incorporate objective airflow measures, validated psychological scales, and harmonized surgical protocols. The development of a core outcome set for ENS research is urgently needed [27].

## Conclusions

Surgical interventions for ENS are associated with statistically and clinically significant improvements in patient-reported outcomes; however, high heterogeneity and limited objective data temper the strength of conclusions.

A high percentag of ENS patients exhibited moderate to severe stress levels compared to other rhinologic conditions,, some even endorsed suicidal ideation, and many showed signs of clinical anxiety or depression. Notable improvements in patients'subjective well-being were found post-surgery. These findings underscore the critical need for integrating mental health screening and support into ENS surgical care protocols.

Future studies should integrate objective physiological assessments and psychological evaluations to enhance comprehensive patient care.

## Data Availability

Not applicable.
